# COVID-19 Pneumonia: Three Thoracic Complications in the Same Patient

**DOI:** 10.3390/diagnostics10070498

**Published:** 2020-07-20

**Authors:** Andrea Borghesi, Carlo Aggiusti, Davide Farina, Roberto Maroldi, Maria Lorenza Muiesan

**Affiliations:** 1 Department of Medical and Surgical Specialties, Radiological Sciences and Public Health, University of Brescia, ASST Spedali Civili of Brescia, Piazzale Spedali Civili 1, 25123 Brescia, Italy; davide.farina@unibs.it (D.F.); roberto.maroldi@unibs.it (R.M.); 2 Department of Clinical and Experimental Sciences, University of Brescia, UOC 2° Medicina, ASST Spedali Civili of Brescia, Piazzale Spedali Civili 1, 25123 Brescia, Italy; carloaggiusti@gmail.com (C.A.); marialorenza.muiesan@unibs.it (M.L.M.)

**Keywords:** COVID-19, computed tomography, acute pulmonary embolism, pulmonary fibrosis, pneumothorax

## Abstract

The most dreaded thoracic complications in patients with coronavirus disease 2019 (COVID-19) are acute pulmonary embolism and pulmonary fibrosis. Both the complications are associated with an increased risk of morbidity and mortality. While acute pulmonary embolism is not a rare finding in patients with COVID-19 pneumonia, the prevalence of pulmonary fibrosis remains unclear. Spontaneous pneumothorax is another possible complication in COVID-19 pneumonia, although its observation is rather uncommon. Herein, we present interesting computed tomography images of the first case of COVID-19 pneumonia that initially developed acute pulmonary embolism and subsequently showed progression toward pulmonary fibrosis and spontaneous pneumothorax.

Subsequent serial chest radiographs showed a progressive reduction in the left-sided pneumothorax. Therefore, considering the reduction in the pneumothorax, chest drainage placement was not indicated by thoracic surgeons.

After 70 days of hospitalization, the patient was discharged and transferred to a rehabilitation center. During hospitalization, the patient was treated with noninvasive ventilation, lopinavir/ritonavir (day 1 to day 10), hydroxychloroquine (day 1 to day 10), empirical antibiotic therapy with azithromycin and ceftriaxone (day 1 to 14), paracetamol, and anticoagulant therapy.

With regard for the noninvasive ventilation, the patient underwent high-flow oxygen therapy with a non-re-breathing reservoir mask (from the day of admission until day 36 post-hospitalization), using a 60% Venturi mask (day 37 to day 63), and then with a nasal cannula (day 64 to discharge).

For the anticoagulant therapy, the patient underwent prophylaxis with low-molecular-weight heparin (from day 1 until day 4 post-hospitalization), therapeutic dose heparin (day 5 to day 47), and then rivaroxaban 20 mg (day 48 to discharge).

At the time of discharge, the patient had no fever but complained of dyspnea on exertion. The left-sided apical pneumothorax became minimal (4 mm thick), and the peripheral oxygen saturation was 95% with a nasal cannula at 2L/min. The blood pressure was 120/65 mmHg. The heart rate and respiratory rate were 99/min and 24/min, respectively.

The most dreaded thoracic complications in patients with COVID-19 pneumonia are acute pulmonary embolism and pulmonary fibrosis. Both the complications are associated with an increased risk of morbidity and mortality. While acute pulmonary embolism is not a rare finding in patients with COVID-19 pneumonia [1,2], the prevalence of pulmonary fibrosis remains unclear [3,4,5]. During previous coronavirus outbreaks, pulmonary fibrosis was reported in up to one-third of patients [3,4,5]. Therefore, given the worldwide spread of COVID-19, a considerable number of cases with associated pulmonary fibrosis are expected.

Spontaneous pneumothorax is another possible complication in COVID-19 pneumonia, although its observation is rather uncommon [6]. Spontaneous pneumothorax is the most common type of pneumothorax. It may occur without any clear cause (primary) or due to underlying pulmonary disease (secondary), especially chronic lung disease [7,8].

Radiological imaging plays a crucial role in the management of patients with COVID-19 pneumonia. Although less sensitive than CT, chest radiography is an effective technique to quantify the severity and monitor the progression of COVID-19 pneumonia [9,10,11,12]. Therefore, chest CT imaging should only be performed in patients with worsening respiratory symptoms or in selected cases (complex or doubtful cases).

In this paper, we have presented interesting CT images of the first case of COVID-19 pneumonia that initially developed acute pulmonary embolism (Figure 1) and subsequently showed progression toward pulmonary fibrosis (Figure 2 and Figure 3) and spontaneous pneumothorax (Figure 3). While several articles have reported the association between COVID-19 pneumonia and acute pulmonary embolism [1,2,13,14,15], only a few papers have described cases of pneumothorax secondary to COVID-19 [16,17,18,19], and none of them have reported its coexistence with pulmonary embolism. In addition, to the best of our knowledge, this report is the first to present unequivocal CT images of progression of COVID-19 pneumonia toward lung fibrosis. COVID-19-associated lung injury and its progression toward pulmonary fibrosis could be the main causative factor for spontaneous pneumothorax in our patient.

This case highlights the importance of considering thoracic complications when the clinical symptoms, respiratory functional parameters, or laboratory tests worsen or do not improve in COVID-19 pneumonia. In such cases, both radiologists and clinicians should be aware of the added value of CT in ruling out complications.

## Figures and Tables

**Figure 1:**
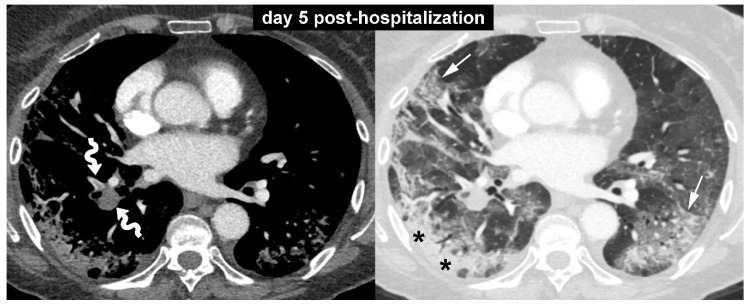
A 73-year-old woman was referred to our emergency department for the evaluation of a 10-day history of fever, dry cough, and shortness of breath. Her medical history was unremarkable except for a previous diagnosis of breast cancer. She had no history of lung comorbidities, such as asthma, chronic obstructive pulmonary disease, or interstitial lung disease. On presentation, the patient exhibited significant breathlessness, and arterial blood gas analysis revealed a pH of 7.51, PCO_2_ of 29 mmHg, PO_2_ of 43 mmHg, and oxygen saturation of 81%. Based on these data, 15 L/min of oxygen was administered through a non-re-breathing reservoir mask. Chest radiography showed bilateral interstitial and alveolar infiltrates in both lungs, greater on the right side. On the same day, the patient underwent nasopharyngeal swab, which confirmed the clinical suspicion of coronavirus disease (COVID-19). The following day the patient was transferred to an intermediate care unit. During the first few days in the hospital, her oxygen saturation was 90–95% with use of the non-re-breathing oxygen mask. On day five post-hospitalization, the patient experienced a sudden deterioration of lung function. Laboratory parameters also showed a significant increase in D-dimer levels (5250 ng/mL; reference value, less than 232 ng/mL). Based on these data, contrast-enhanced computed tomography (CT) scan was performed. The contrast-enhanced CT images with mediastinal and lung window setting (Figure 1) showed filling defects in some branches of the pulmonary artery (curved arrows) and bilateral ground-glass opacities (arrows) associated with areas of consolidation (asterisks). No evidence of pulmonary fibrosis was found in this initial CT examination.

**Figure 2:**
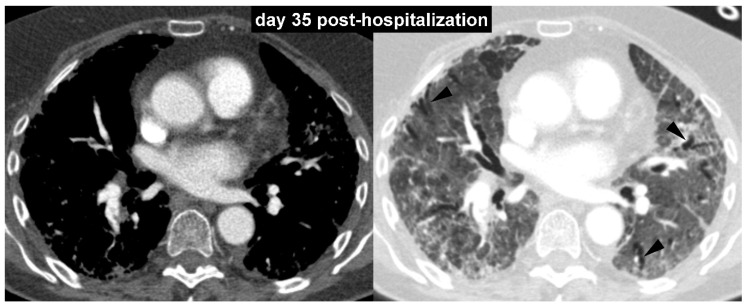
To monitor parenchymal lung disease, serial chest radiographs with different time intervals were obtained. However, to evaluate the response of pulmonary vascular disease to anticoagulant therapy, a further contrast-enhanced CT scan was performed after one month (day 35 post-hospitalization). The follow-up contrast-enhanced CT images with mediastinal and lung window setting (Figure 2) showed significant regression of the pulmonary embolism and lung abnormalities (both consolidations and ground-glass opacities). However, initial signs of lung architectural distortion with some traction bronchiectasis (arrowheads) were observed.

**Figure 3:**
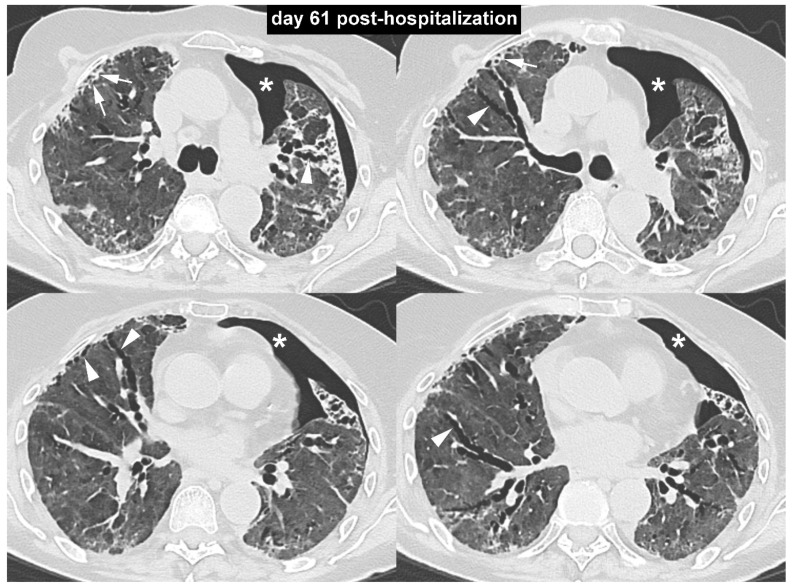
The subsequent bedside radiographic follow-up showed a trend toward gradual reduction in pulmonary opacities. On day 61 post-hospitalization, owing to persistent breathlessness and the need for high-flow oxygen therapy, an unenhanced high-resolution chest CT scan was performed. The high-resolution CT images with lung window setting (Figure 3) demonstrated the persistence of a diffuse increase in lung density with ground-glass appearance, associated with peripheral irregular reticular opacities (arrows) and several traction bronchiectasis (arrowheads). These findings were consistent with the diagnosis of pulmonary fibrosis. In addition, an unexpected left-sided spontaneous pneumothorax was noted (asterisks).

## References

[1] Grillet F., Behr J., Calame P., Aubry S., Delabrousse E. (2020). Acute Pulmonary Embolism Associated with COVID-19 Pneumonia Detected by Pulmonary CT Angiography. Radiology.

[2] Faggiano P., Bonelli A., Paris S., Milesi G., Bisegna S., Bernardi N., Curnis A., Agricola E., Maroldi R. (2020). Acute pulmonary embolism in COVID-19 disease: Preliminary report on seven patients. Int. J. Cardiol..

[3] Spagnolo P., Balestro E., Aliberti S., Cocconcelli E., Biondini D., Casa G.D., Sverzellati N., Maher T.M. (2020). Pulmonary fibrosis secondary to COVID-19: A call to arms?. Lancet Respir. Med..

[4] Gentile F., Aimo A., Forfori F., Catapano G., Clemente A., Cademartiri F., Emdin M., Giannoni A. (2020). COVID-19 and risk of pulmonary fibrosis: The importance of planning ahead. Eur. J. Prev. Cardiol..

[5] Lechowicz K., Drożdżal S., Machaj F., Rosik J., Szostak B., Zegan-Barańska M., Biernawska J., Dabrowski W., Rotter I., Kotfis K. (2020). COVID-19: The Potential Treatment of Pulmonary Fibrosis Associated with SARS-CoV-2 Infection. J. Clin. Med..

[6] Chen N., Zhou M., Dong X., Qu J., Gong F., Han Y., Qiu Y., Wang J., Liu Y., Wei Y. (2020). Epidemiological and clinical characteristics of 99 cases of 2019 novel coronavirus pneumonia in Wuhan, China: A descriptive study. Lancet.

[7] Onuki T., Ueda S., Yamaoka M., Sekiya Y., Yamada H., Kawakami N., Araki Y., Wakai Y., Saito K., Inagaki M. (2017). Primary and Secondary Spontaneous Pneumothorax: Prevalence, Clinical Features, and In-Hospital Mortality. Can. Respir. J..

[8] Hallifax R.J., Goldacre R., Landray M.J., Rahman N.M., Goldacre M.J. (2018). Trends in the Incidence and Recurrence of Inpatient-Treated Spontaneous Pneumothorax, 1968–2016. JAMA.

[9] Borghesi A., Maroldi R. (2020). COVID-19 outbreak in Italy: Experimental chest X-ray scoring system for quantifying and monitoring disease progression. Radiol. Med..

[10] Borghesi A., Zigliani A., Masciullo R., Golemi S., Maculotti P., Farina D., Maroldi R. (2020). Radiographic severity index in COVID-19 pneumonia: Relationship to age and sex in 783 Italian patients. Radiol. Med..

[11] Borghesi A., Zigliani A., Golemi S., Carapella N., Maculotti P., Farina D., Maroldi R. (2020). Chest X-ray severity index as a predictor of in-hospital mortality in coronavirus disease 2019: A study of 302 patients from Italy. Int. J. Infect Dis..

[12] Vancheri S.G., Savietto G., Ballati F., Maggi A., Canino C., Bortolotto C., Valentini A., Dore R., Stella G.M., Corsico A.G. (2020). Radiographic findings in 240 patients with COVID-19 pneumonia: Time-dependence after the onset of symptoms. Eur. Radiol..

[13] Gervaise A., Bouzad C., Peroux E., Helissey C. (2020). Acute pulmonary embolism in non-hospitalized COVID-19 patients referred to CTPA by emergency department. Eur. Radiol..

[14] Cellina M., Oliva G. (2020). Acute pulmonary embolism in a patient with COVID-19 pneumonia. Diagn. Interv. Imaging.

[15] Brogna B., Brogna C., Martino A., Minichiello S., Romeo D.M., Romano P., Bignardi E., Mazza E.M., Musto L. (2020). SARS-CoV-2 Infection with Different Radiological Insights. Diagnostics.

[16] Wang W., Gao R., Zheng Y., Jiang L. (2020). COVID-19 with spontaneous pneumothorax, pneumomediastinum and subcutaneous emphysema. J. Travel Med..

[17] Ucpinar B.A., Sahin C., Yanc U. (2020). Spontaneous pneumothorax and subcutaneous emphysema in COVID-19 patient: Case report. J. Infect Public Health.

[18] Flower L., Carter J.L., Rosales Lopez J., Henry A.M. (2020). Tension pneumothorax in a patient with COVID-19. BMJ Case Rep..

[19] Spiro J.E., Sisovic S., Ockert B., Böcker W., Siebenbürger G. (2020). Secondary tension pneumothorax in a COVID-19 pneumonia patient: A case report. Infection.

